# Tetramerization of STAT5 regulates monocyte differentiation and the dextran sulfate sodium-induced colitis in mice

**DOI:** 10.3389/fimmu.2023.1117828

**Published:** 2023-04-20

**Authors:** Kelly L. Monaghan, Wen Zheng, Halima Akhter, Lei Wang, Amanda G. Ammer, Peng Li, Jian-Xin Lin, Gangqing Hu, Warren J. Leonard, Edwin C. K. Wan

**Affiliations:** ^1^ Department of Microbiology, Immunology, and Cell Biology, West Virginia University, Morgantown, WV, United States; ^2^ Department of Computer Science and Electrical Engineering, West Virginia University, Morgantown, WV, United States; ^3^ Microscope Imaging Facility, West Virginia University, Morgantown, WV, United States; ^4^ Laboratory of Molecular Immunology and the Immunology Center, National Heart, Lung, and Blood Institute, National Institutes of Health, Bethesda, MD, United States; ^5^ Bioinformatics Core, West Virginia University, Morgantown, WV, United States; ^6^ Department of Neuroscience, West Virginia University, Morgantown, WV, United States; ^7^ Rockefeller Neuroscience Institute, West Virginia University, Morgantown, WV, United States

**Keywords:** STAT5 tetramers, monocytes, GM-CSF, colitis, arginase I

## Abstract

In response to external stimuli during immune responses, monocytes can have multifaceted roles such as pathogen clearance and tissue repair. However, aberrant control of monocyte activation can result in chronic inflammation and subsequent tissue damage. Granulocyte-macrophage colony-stimulating factor (GM-CSF) induces monocyte differentiation into a heterogenous population of monocyte-derived dendritic cells (moDCs) and macrophages. However, the downstream molecular signals that dictate the differentiation of monocytes under pathological conditions is incompletely understood. We report here that the GM-CSF-induced STAT5 tetramerization is a critical determinate of monocyte fate and function. Monocytes require STAT5 tetramers to differentiate into moDCs. Conversely, the absence of STAT5 tetramers results in a switch to a functionally distinct monocyte-derived macrophage population. In the dextran sulfate sodium (DSS) model of colitis, STAT5 tetramer-deficient monocytes exacerbate disease severity. Mechanistically, GM-CSF signaling in STAT5 tetramer-deficient monocytes results in the overexpression of arginase I and a reduction in nitric oxide synthesis following stimulation with lipopolysaccharide. Correspondingly, the inhibition of arginase I activity and sustained supplementation of nitric oxide ameliorates the worsened colitis in STAT5 tetramer-deficient mice. This study suggests that STAT5 tetramers protect against severe intestinal inflammation through the regulation of arginine metabolism.

## Introduction

Monocytes are innate immune cells that have instrumental roles in pathogen clearance, the amplification of inflammatory responses, and the resolution of tissue damage (1). The fate of monocyte differentiation is regulated by a combination of external stimuli, which have been extensively studied *in vitro* (2–4). Upon activation, monocytes differentiate into a heterogenous population of monocyte-derived cells (MDCs) that is comprised of monocyte-derived dendritic cells (moDCs) and macrophages. moDCs predominantly facilitate antigen presentation and subsequent T-cell expansion, whereas macrophages express proinflammatory cytokines and promote the phagocytosis of apoptotic cells. In some instances, macrophages can also facilitate tissue repair and angiogenesis. These monocyte-derived macrophages are conventionally characterized using a binary classification method that defines them as either proinflammatory “M1” or immunomodulatory “M2” cells (5). This characterization is often based on the expression of “M1/M2” markers that are differentially expressed by these cells. However, the functional dichotomy of M1/M2 macrophages may not apply under inflammatory conditions given that it does not account for the nuanced signals that macrophages receive under these context-dependent conditions. Thus, there is a need to elucidate the molecular mechanisms, downstream of cytokine signaling, that dictate the fate of monocyte differentiation and function.

The cytokine granulocyte-macrophage colony-stimulating factor (GM-CSF) plays a critical role in regulating autoimmune-associated inflammation and chronic inflammation (6, 7). GM-CSF induces the terminal differentiation of monocytes into moDCs and macrophages, with a proclivity toward moDCs (8–11). GM-CSF-mediated activation of monocytes is generally thought to promote the pathogenesis of autoimmune diseases, such as rheumatoid arthritis and multiple sclerosis (11, 12). However, animal studies demonstrate that GM-CSF protects mice from experimental models of inflammatory bowel disease (IBD), which includes Crohn’s disease and ulcerative colitis. In addition, early clinical studies suggest that GM-CSF administration decreases disease severity in patients with active Crohn’s disease (13–16). However, the mechanisms by which GM-CSF modulates colitis severity are not completely understood. Multiple signaling pathways, including the JAK2-STAT5, MAPK, and PI3K-AKT pathways, are activated upon GM-CSF stimulation. Among which the transcription factor STAT5 is the major signaling mediator that regulates the GM-CSF-induced responses during monocyte differentiation (7, 17). Upon activation, STAT5 proteins undergo functional dimerization *via* SH2 domain-phosphotyrosine interactions. Interactions between two STAT5 dimers *via* their N-terminal regions (N-domains) result in tetramerization of STAT5. STAT5 tetramers can bind to lower affinity non-consensus γ interferon activated sequence (GAS) motifs that are optimally spaced, allowing for the regulation of target genes (18–20). The physiological functions of STAT5 tetramers can be investigated using the *Stat5a*-*Stat5b* N-domain double knock-in (DKI) mice, in which substitutions at I28A and F81A in STAT5A and STAT5B prevent STAT5 tetramerization (19). Using this mouse strain, we recently demonstrated that STAT5 tetramers promote autoimmune-mediated neuroinflammation *via* the regulation of CCL17 expression in MDCs. CCL17 in turn promotes the extravasation of pathogenic T-helper 17 (Th17) cells from the meninges into the spinal cord parenchyma (21). Here, we report that STAT5 tetramers also play a critical role in controlling the fate of monocyte differentiation. GM-CSF-mediated activation of STAT5 tetramers promotes monocyte differentiation into moDCs but suppresses their differentiation into macrophages. In addition, contrary to their pathogenic role in neuroinflammation, STAT5 tetramers delay DSS-induced colitis in mice by suppressing the expression of arginase I by MDCs. This study highlights opposing context-dependent effects of STAT5 tetramers in inflammatory diseases.

## Results

### STAT5 tetramers promote moDC but suppress macrophage differentiation

**Figure 1:**
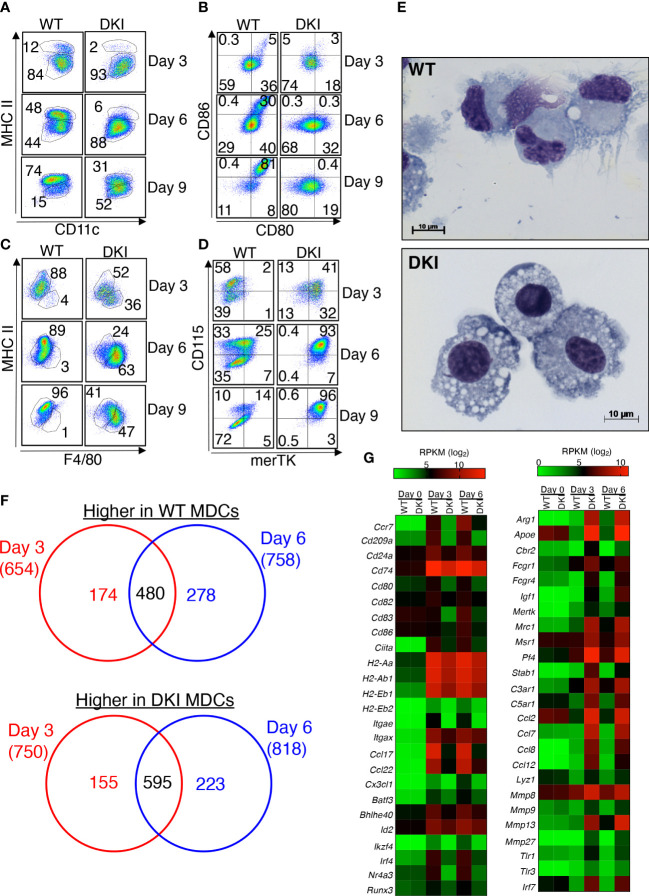
STAT5 Tetramers Promote moDC but Suppress Macrophage Differentiation. **(A-G)** Bone marrow-derived monocytes were isolated from WT and DKI mice. Monocytes were stimulated with 20 ng/ml GM-CSF for 3, 6, or 9 d. On either day 3, 6, or 9 differentiated cells were harvested for downstream analysis. **(A-D)** Representative dot plots showing the expression of CD11c and MHCII **(A)**, CD80 and CD86 **(B)**, F4/80 and MHCII **(C)**, and merTK and CD115 **(D)** following 3, 6, and 9 days of differentiation. Representative dot plots are from 3 independent experiments. **(E)** Representative Wright-Giemsa staining on differentiated WT and DKI MDCs following 8 d differentiation from 3 independent experiments. **(F, G)** RNA was isolated from WT and DKI unstimulated monocytes (day 0) and MDCs following 3 and 6 days of differentiation with GM-CSF. **(F)** Venn diagram comparing STAT5 tetramer-dependent genes that were differentially expressed following 3 and 6 d of differentiation. **(G)** Heatmap showing the expression levels (RPKM; log_2_) of selected moDC-associated genes (left) and macrophage-associated genes (right) in WT and DKI MDCs. **(F, G)** Data are combined from 3 independent experiments.

We first sought to investigate the role of STAT5 tetramers in monocyte differentiation. WT and DKI monocytes were stimulated *in vitro* with 20 ng/mL GM-CSF, and the expression of moDC and macrophage lineage markers was determined on days 3, 6, and 9. The purity of monocytes isolated from the bone marrow was greater than 85% (**Supplemental Figure 1A**). As expected, GM-CSF induced the differentiation of monocytes into moDCs, which were characterized as CD11c+ MHCII^hi^ CD80^hi^ CD86^hi^ (**Figures 1A, B**). However, upon GM-CSF stimulation, the DKI monocytes exhibited reduced expression of MHCII, CD80 and CD86 (**Figures 1A, B**), but increased expression of the macrophage-associated markers F4/80, CD115, and merTK (**Figures 1C, D**). In addition, when stimulated with GM-CSF for 8 days, the cells in the WT culture had a dendritic cell morphology, whereas the cells in the DKI culture had a macrophage morphology (**Figure 1E**). We asked whether the effect of STAT5 tetramers on monocyte differentiation is dependent of the dose of GM-CSF. We stimulated WT and DKI monocytes with 2 or 5 ng/mL GM-CSF and found that WT monocytes maintained the preference to express markers for moDCs, whereas DKI monocytes were skewed to express the macrophage-associated markers. However, the percent of differentiating monocytes expressing these markers were less compared with 20 ng/mL GM-CSF stimulation (**Supplemental Figures 1B-F**). Thus, STAT5 tetramers appear to strongly regulate the proportion of monocytes that differentiate into moDCs compared to macrophages. We posited that STAT5 tetramers promote the differentiation of monocytes into a genetically distinct population of cells. To investigate this, the differential gene expression in WT and DKI monocytes was assessed following 3 and 6 days of differentiation with GM-CSF. Multidimensional scaling analysis shows that the gene expression profiles of unstimulated WT and DKI monocytes were comparable, but there were substantial differences in WT and DKI MDCs following GM-CSF stimulation (**Supplemental Figure 2A** and **Supplemental Dataset 1**). STAT5 tetramers predominantly act as transcriptional activators in T cells (19). In MDCs, the expression of 654 and 758 genes were reduced in DKI MDCs compared to WT MDCs at 3 and 6 days following GM-CSF stimulation, respectively (**Figure 1F** and **Supplemental Datasets 2-4**). Some of these STAT5 tetramer-dependent genes, including signature genes for DCs (*Ccr7, Cd209a, Ccl17,* and *Ccl22* ) and genes that control antigen presentation and co-stimulation (*Cd74, Cd80, Cd82, CD83, Cd86, H2-Aa, H2-Ab1*) were regulated by STAT5 tetramers (22-24) (**Figure 1G** and **Supplemental Figure 2B**). In addition, the expression of several transcriptional factors known to regulate the differentiation or function of DCs, such as *Batf3, Id2, Ikzf4,* and *Irf4*, were also regulated by STAT5 tetramers (**Figure 1G**). These data suggest that STAT5 tetramers may initiate the moDC differentiation program in monocytes that involves multiple transcriptional pathways. Intriguingly, the expression of many genes (750 on day 3 and 818 on day 6) was increased in the absence of STAT5 tetramers, including *Fcgr1, Fcgr4, Mrc1, Pf4,* and *Stab1*, which are signature genes for macrophages (**Figures 1F, G** and **Supplemental Figure 2B and Supplemental Dataset 2-4**). Correspondingly, Geneset analysis utilizing the Immunological Genome Project database (ImmGen) confirmed the DC phenotype of the WT MDCs and the macrophage phenotype of the DKI MDCs (**Supplemental Figure 2C**). In addition to STAT5, GM-CSF also activates MAPK and PI3K-AKT signaling pathways (17). We used Gene Set Enrichment Analysis (GSEA) to determine the effect of STAT5 tetramer deficiency on the activation of these pathways and found that STAT5 tetramer deficiency did not affect genes that regulated by MAPK and PI3K-AKT signaling (**Supplemental Figure 2D**). The WT and DKI MDCs exhibited comparable levels of STAT5 phosphorylation and GM-CSF receptor expression, suggesting that the observed phenotypic differences were due to altered transcriptional regulation (**Supplemental Figure 3A, B**). In fact, the expression of GM-CSFRα and the common β chain (β_c_) was higher in DKI MDCs compared to the WT MDCs (**Supplemental Figure 3B**). A recent study demonstrated that moDCS, monocyte-derived macrophages, and conventional dendritic cells arise from a distinct and heterogenous population of Ly6Chi monocytes that have differential expression of the markers Flt3, CD11c, PU.1, and MHCII (25). However, the proportions of these progenitor cells were similar in the WT and DKI bone marrow (**Supplemental Figure 4A-C**). Moreover, the percent of CD11b^+^ Ly6C^+^ CCR2^+^ monocytes was comparable in WT and DKI bone morrow (**Supplemental Figure 4D**). In addition, the percent of splenic CD11c^+^ conventional dendritic cells (cDCs), which can be further categorized into CD4^+^ CD8^-^, CD4^-^ CD8^+^, or CD4^-^ CD8^-^ subtypes, was comparable in WT and DKI mice (**Supplemental Figure 4E, F**). These data suggest that STAT5 tetramers do not regulate the development of monocytes and cDCs from their respective progenitor cells. Furthermore, the expression level of F4/80 and merTK in macrophages isolated from the lungs, spleen, and peritoneal cavity of WT and DKI mice was comparable (**Supplemental Figure 5A–C**). However, the total number of F4/80^+^ macrophages was lower in DKI mice than in WT mice (**Supplemental Figure 5A**). Taken together, GM-CSF-mediated STAT5 tetramerization in monocytes promotes moDC but inhibits macrophage differentiation.

### STAT5 tetramers control the functions of MDCs

**Figure 2:**
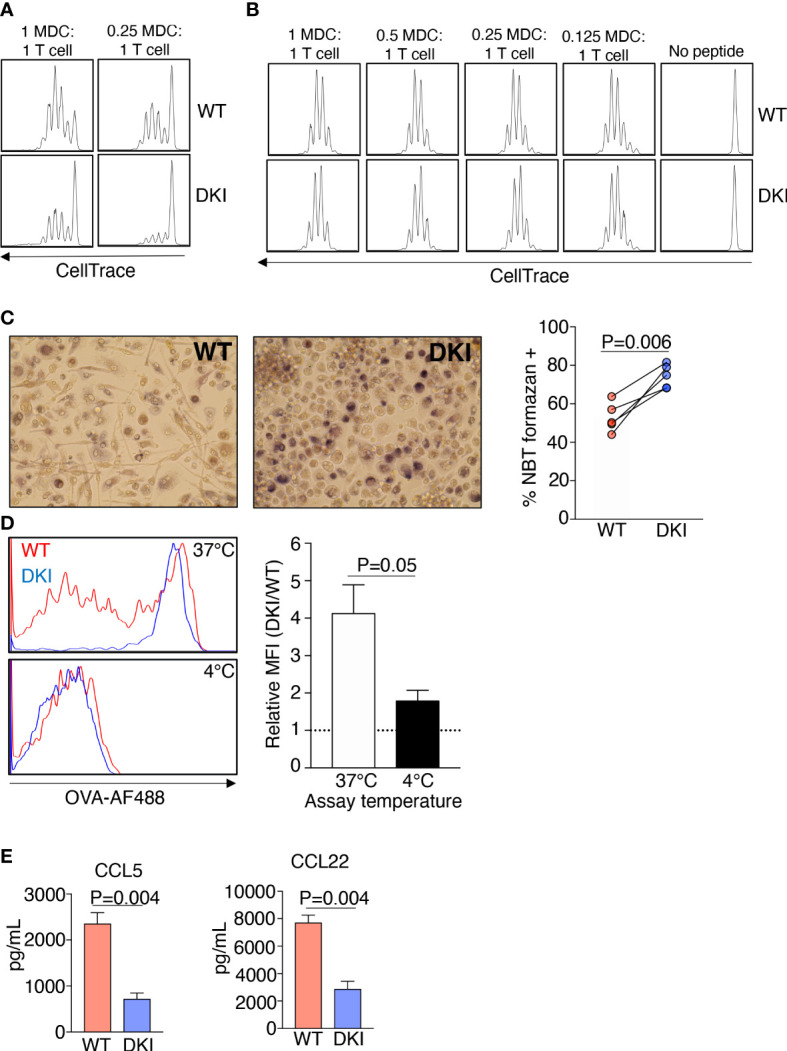
STAT5 Tetramers Control the Functions of MDCs. **(A-E)** WT and DKI bone marrow monocyte were differentiated for 8 d with GM-CSF. **(A, B)** Naïve OT-II CD4^+^ T cells were labeled with CellTrace Violet and were co-cultured with WT or DKI MDCs at different ratios in the presence ovalbumin protein **(A)** or OVA _(323-339)_ peptide **(B)** for 72 h. Representative histograms showing the dilution of CellTrace Violet. Data are from 3 independent experiments. **(C)** Percent of WT and DKI MDCs containing NBT formazan deposits following 1 h stimulation with LPS and 15 min incubation with nitro blue tetrazolium. Representative images are shown. Data are from 5 independent experiments. **(D)** Representative histogram showing the ability WT and DKI MDCs to phagocytose fluorescently conjugated ovalbumin. Incubation at 4°C was used as a negative control to omit any signal from fluorescently conjugated ovalbumin bound to the surface of the cells. DKI MFI relative to WT MFI is shown. Data are combined from 5 independent experiments. **(E)** Expression of CCL5 and CCL22 in the culture supernatants of WT and DKI MDCs. Data are combined from 3 independent experiments. **(C–E)** Data shown are mean ± SEM. Statistical significance is indicated by the P-value with NS meaning no statistical significance. **(C)** Paired *t* test. **(D, E)** Unpaired *t* test.

Having established that GM-CSF-mediated STAT5 tetramers regulate the genetic identity and the expression of surface markers on MDCs, we sought to determine whether STAT5 tetramers regulated the function of MDCs. Dendritic cells predominantly process and present antigens, whereas macrophages clear pathogens by phagocytosis and the production of oxidative bursts (26). To assess the antigen processing and presenting ability of the WT and DKI MDCs, these cells were co-cultured with ovalbumin 323-339 peptide (OVA_323-339_)-T cell receptor restricted CD4^+^ T cells (OT-II) in the presence of either 2 μg/mL OVA protein or 2, 5, or 20 μg/mL OVA_323-339_ peptide. WT MDCs exhibited an increased ability to induce the proliferation of CD4^+^ T cells compared to DKI MDCs when co-cultured in the presence of ovalbumin protein (**Figure 2A**). However, the extent of CD4^+^ T cell proliferation was comparable when OVA_323-339_ peptide was added, regardless of whether the CD4^+^ T cells were co-cultured with WT or DKI MDCs, or the concentration of OVA_323-339_peptide (**Figure 2B** and **Supplemental Figure 6**). These data suggest that STAT5 tetramers promote antigen processing but not antigen presentation in MDCs. In contrast, DKI MDCs were more equipped to phagocytose fluorescent-conjugated OVA and to produce intracellular superoxide following LPS stimulation (**Figures 2C, D**). In addition, WT MDCs had higher expression of the moDC-associated chemokines CCL5, CCL17, and CCL22 both at the level of mRNA (**Figure 1G**) and protein [**Figure 2E** and ref (21)]. These data indicate that GM-CSF activated STAT5 tetramers have the capacity to regulate the differentiation of monocytes into a functionally distinct population of moDCs. Conversely, the absence of functional STAT5 tetramers results in a switch to a functionally distinct monocyte-derived macrophage population.

### STAT5 tetramers in monocytes regulate the severity of colitis

**Figure 3:**
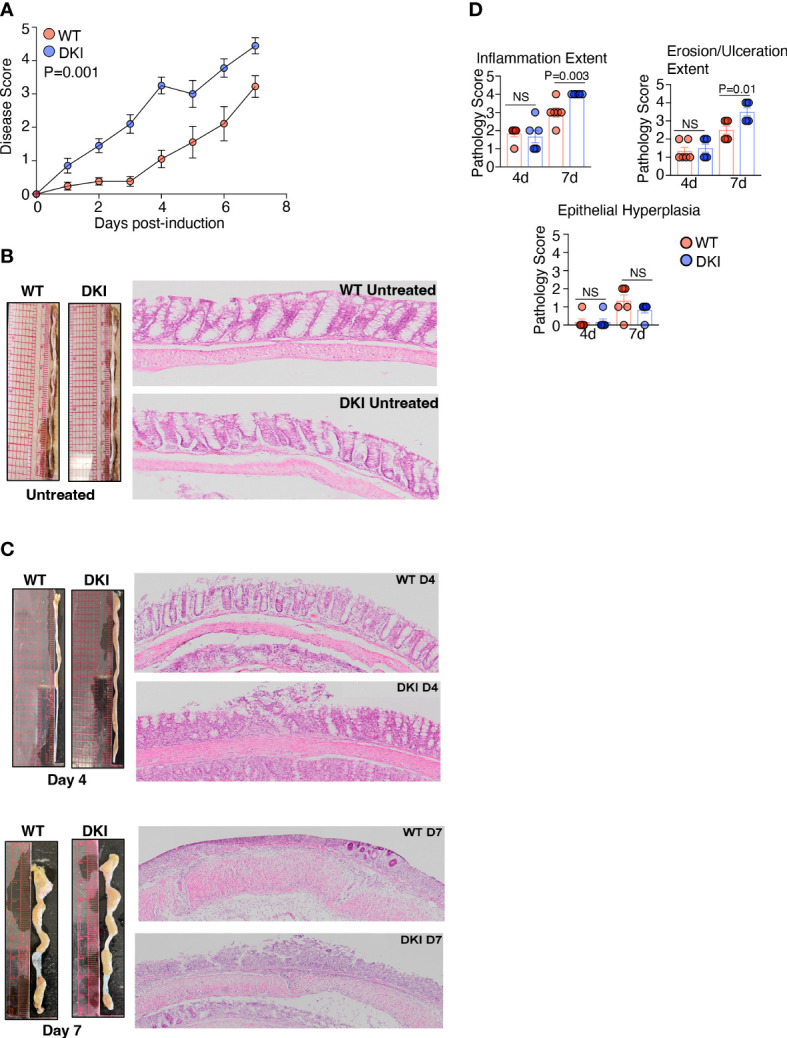
STAT5 Tetramers Prevent Serve Colitis Induced By DSS. **(A-C)** Colitis was induced in WT and DKI mice *via* the administration of 1% DSS to the drinking water. Colons were harvested on either day 4 or 7 post colitis induction. **(A)** Disease scores. n=21 for WT and 20 for DKI from 4 independent experiments. **(B, C)** Representative images and H&E staining of colons on **(B)** untreated mice (n=10) or **(C)** mice treated with DSS for 4 or 7 days. In **(C)**, Data are from 2 independent experiments per timepoint with n=6. **(D)** Pathological assessment of inflammation extent (total area of the colon presenting with inflammation), ulceration/erosion extent (total area of the colon presenting with ulceration/erosion), and epithelial hyperplasia. Data are from 2 independent experiments per timepoint with n=6. **(A, D)** Data shown are mean ± SEM. Statistical significance is indicated by the P-value with NS meaning no statistical significance. **(A)** Area under the curve with unpaired *t* test. **(D)** Unpaired *t* test.

The importance of STAT5 signaling in intestinal inflammation has been investigated previously. These studies have demonstrated that STAT5 activation impedes colitis severity, possibly by regulating intestinal barrier integrity (27–29). Thus, we sought to determine whether STAT5 tetramers regulate the pathogenesis of DSS-induced colitis. Colitis was induced in WT and DKI mice by administering 1% DSS to the drinking water, and disease severity as assessed on days 4 and 7. The DKI mice exhibited more severe colitis compared to the WT mice (**Figure 3A**), as indicated by an increased disease score, which was observed as early as 1 day following DSS administration. The disease score was determined by assessing the extent of water retention in the stool, the presence of blood in the stool, and the extent of rectal bleeding (**Supplemental Figure 7A**). Weight loss and the shortening of colon length, which are parameters often used to evaluate colitis severity, were comparable in WT and DKI mice following DSS administration (**Supplemental Figures 7B, C**). This is likely because a relatively low percent of DSS was used within a short evaluation period. Pathological assessment determined that there were no intrinsic differences in the WT and DKI colons without DSS administration (**Figure 3B**). However, on day 4 following DSS administration, DKI colons had increased colon diameter along with distinct, focal areas of increased inflammation, ulceration, and epithelial cell erosion, which extended more diffusely throughout the colon on day 7 compared to WT colons. (**Figures 3C, D**). Minimal and comparable levels of epithelial hyperplasia were observed in the WT and DKI colons (**Figure 3D**). Taken together, these data suggest that STAT5 tetramers regulate the severity of DSS-induced colitis.

**Figure 4:**
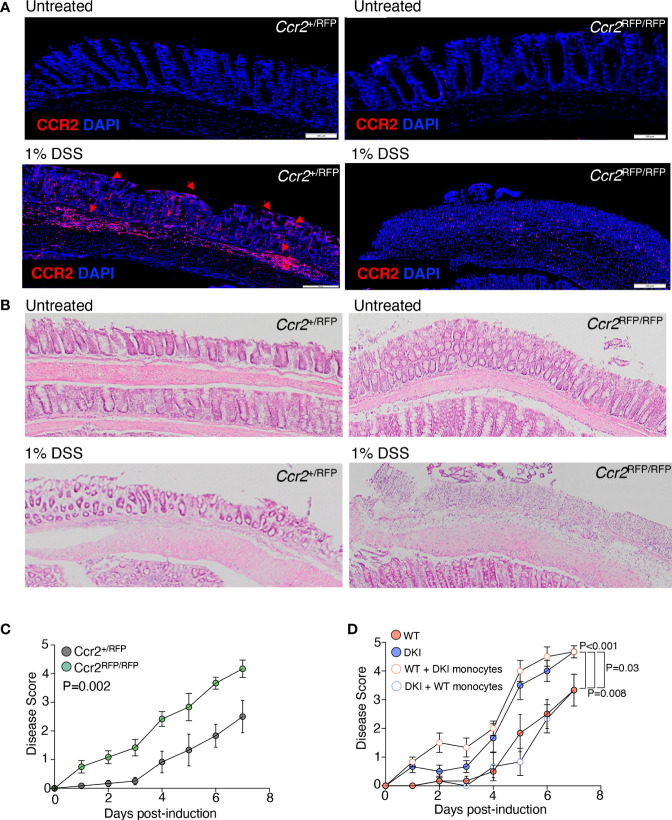
STAT5 Tetramers in Monocytes Regulate the Severity of Colitis. **(A, B)** Colon sections *Ccr2* ^+/RPF^ and *Ccr2* ^RFP/RFP^ mice with and without DSS treatment. **(A)** Representative images showing monocyte migration to the site damage in the epithelial monolayer following treatment with 1% DSS (red arrows). **(B)** Representative H&E-stained colon sections. Data are from 2 independent experiments with n=6. **(C)** *Ccr2* ^+/RFP^ and *Ccr2* ^RFP/RFP^ mice were treated with 1% DSS for 7 d. Disease scores are shown. Data are from 4 independent experiments with n=13 for WT and 12 for DKI. **(D)** WT and DKI monocytes were transferred into WT and DKI recipient mice on days 0 and 2 following treatment with 1% DSS. Colitis disease scores are shown. Data are from 2 independent experiments with n=6. **(C, D)** Data shown are mean ± SEM. Statistical significance is indicated by the P-value with NS meaning no statistical significance. **(C)** Area under the curve with unpaired *t* test. **(D)** Area under the curve with one way ANOVA and Tukey’s test for multiple comparisons.

Previous studies have demonstrated that colitis induced by DSS can promote the recruitment of inflammatory monocytes to the colon, which can differentiate into moDCs and macrophages. In addition, the ablation of intestinal mononuclear phagocytes exacerbates colitis severity, which is associated with increased epithelial cell injury (30), suggesting that monocytes confer protection against severe colitis. Therefore, we speculated that STAT5 tetramerization in monocytes ameliorates colitis severity. The recruitment of monocytes following DSS administration was first determined using *Ccr2* ^+/RFP^ mice, which have a gene encoding red fluorescent protein (RFP) inserted into *Ccr2* locus (**Figure 4A**). In the heterozygous *Ccr2* ^+/RFP^ mice, the CCR2^+^ monocytes migrated to the damaged epithelial monolayer. The migration of monocytes was substantially reduced in homozygous *Ccr2* ^RFP/RFP^ mice, in which CCR2 is not expressed, indicating that the CCL2-CCR2 signaling is required for monocyte recruitment (**Figure 4A**). Interestingly, the homozygous CCR2-deficient mice exhibited earlier onset and more severe colitis compared to their heterozygous counterparts, suggesting that monocytes ameliorate DSS-induced colitis severity during the early phase of disease (**Figures 4B, C**). Importantly, adoptive transfer of WT CCR2^+^ monocytes to DKI mice delayed the onset and ameliorated the severity of colitis following DSS administration, and the disease course in DKI mice the received WT monocytes was comparable to the WT mice (**Figure 4D**). These results indicate that STAT5 tetramers in monocytes protect against DSS-induced colonic damage. Surprisingly, the adoptive transfer of DKI CCR2^+^ monocytes to WT mice exacerbated colitis severity, with a similar disease progression to the DKI mice (**Figure 4D**). These data strongly suggest that STAT5 tetramers in monocytes not only protected the mice from colonic epithelial damage, but the loss of STAT5 tetramers in monocytes increased their pathogenicity.

### Arginase I production by DKI monocytes promotes colitis severity

**Figure 5:**
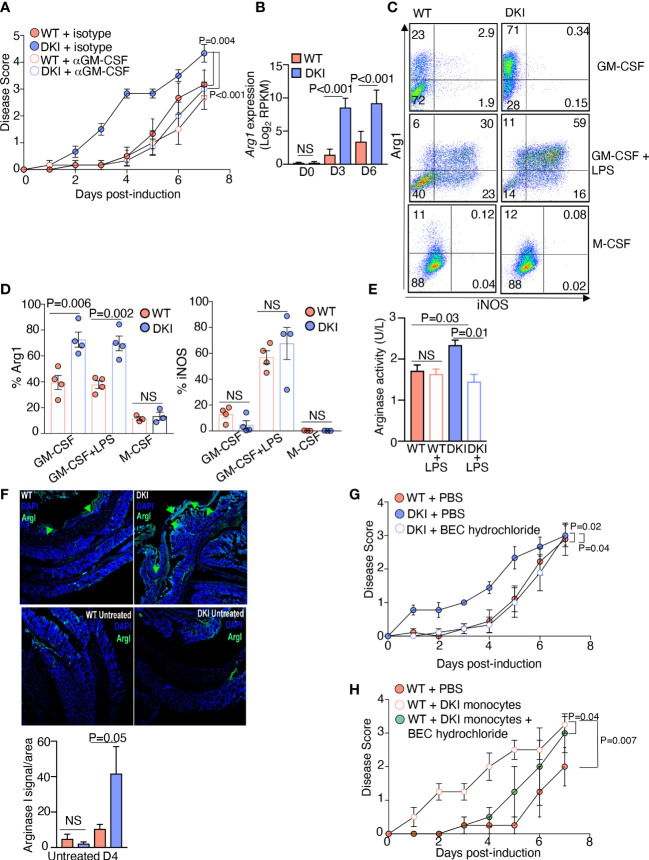
Arginase I Production by DKI Monocytes Promotes Colitis Severity. **(A)** Anti-GM-CSF or an isotype control antibody were administered to WT and DKI mice on days 0, 2, and 4 following 1% DSS administration. Mice were treated with DSS for 7 days. Disease scores are shown. Data are from 3 independent experiments with n=6. **(B)** Gene expression level of *Arg1* in WT and DKI MDCs following 0, 3, and 6 d of differentiation with GM-CSF. Data are combined from 3 independent experiments. **(C,D)** Expression of iNOS and arginase I in WT and DKI MDCs following 8 d of differentiation with either GM-CSF or M-CSF. Some MDCs were differentiated with GM-CSF, then stimulated with LPS for 16 h. Representative dot plots **(C)** and the combined results **(D)** from 4 independent experiments are shown. **(E)** Arginase I activity in WT and DKI MDCs differentiated with GM-CSF for 8 d. Some MDCs were stimulated with LPS for 16h. Activity was measured for 20 min. Data are combined from 3 independent experiments. Shown is the activity of arginase in unit per liter (U/L), defined by the amount of arginase that generates 1 μmol of H_2_O_2_ per minute at pH 8 at 37^°^C. **(F)** Representative images showing the expression of arginase I in WT and DKI colons with or without 4 d of DSS treatment. Data are from 2 independent experiments with n=6. **(G)** Disease scores from WT mice, DKI mice, and DKI mice treated with BEC hydrochloride 0, 1, and 2 d following colitis induction. Data is combined from 3 independent experiments with n=9. **(H)** Disease scores from WT mice, WT recipient mice that received DKI monocytes on days 0 and 2 post colitis induction, and WT mice recipient mice that received DKI monocytes on days 0 and 2 post colitis induction and BEC hydrochloride on days 0, 1, and 2 post colitis induction. Data are from 2 independent experiments with n=4. **(A, B, D-H)** Data shown are mean ± SEM. Statistical significance is indicated by the P-value with NS meaning no statistical significance. **(A, G, H)** Area under the curve with one way ANOVA and Tukey’s test for multiple comparisons. **(B, D, E)** Unpaired *t* test. **(F)** Paired *t* test.

Having determined that the STAT5 tetramer-dependent regulation of monocyte function is contingent on GM-CSF signaling, we posited that the increased pathogenicity of the DKI monocytes was due to dysregulated GM-CSF signaling in the absence of STAT5 tetramers. Administration of a neutralizing antibody against GM-CSF did not alter colitis severity in WT mice; however, it significantly delayed the onset and ameliorated the severity of colitis in DKI mice to an extent comparable to WT mice (**Figure 5A**). These data strongly suggest that in the absence of STAT5 tetramers, GM-CSF signaling exacerbated DSS-induced colitis, likely through its effects on monocytes. We next sought to identify the downstream mediator of GM-CSF signaling that was promoting colitis severity in the absence of STAT5 tetramers. Analysis of the differential gene expression in WT and DKI MDCs following GM-CSF stimulation demonstrated that there was a significant up-regulation in the expression of the *Arg1* gene (**Figure 1G**), which encodes arginase I, in DKI MDCs compared to WT MDCs (**Figure 5B**). Previous studies demonstrated that the genetic deletion of arginase I or chemical inhibition of arginase activity ameliorates colitis induced by DSS (31, 32). Additionally, biopsies from patients with both Crohn’s disease and ulcerative colitis have been found to have higher mRNA levels of arginase I, suggesting a potentially pathogenic role for arginase I in IBD (32). Therefore, we speculated that GM-CSF-induced arginase I expression in STAT5 tetramer-deficient monocytes exacerbates colitis. GM-CSF induced higher protein level (**Figures 5C, D**) and activity of arginase I (**Figure 5E**) in STAT5 tetramer-deficient monocytes as compared to WT monocytes. However, GM-CSF did not regulate the expression of inducible nitric oxide synthase (iNOS) in monocytes without LPS stimulation, which is independent of STAT5 tetramer signaling (**Figures 5C, D**). Conversely, LPS did not regulate the expression of arginase I in monocytes (less than 2% Arg1^+^ cells) without prior GM-CSF stimulation (**Supplemental Figure 8**). In addition, the expression of arginase I was comparable in WT and DKI monocytes following M-CSF stimulation (**Figures 5C, D**). These data suggest that the regulation of arginase I by STAT5 tetramers was GM-CSF-specific. Interestingly, after treatment with DSS, there was a significant increase in the expression of arginase I in the colons of DKI mice compared to WT mice. In the DKI colons, arginase I was present in areas where epithelial cell damage and erosion were observed (**Figure 5F**). The administration of an arginase I inhibitor, BEC hydrochloride, ameliorated DSS-induced colitis in DKI mice to a disease severity level comparable to that observed in WT mice (**Figure 5G**), suggesting that the exacerbated colitis in DKI mice was caused by an increase in arginase I activity. To further investigate whether the increase in arginase I activity in MDCs was responsible for the exacerbated colitis, DSS was administered to WT mice following DKI monocyte transfer and treatment with BEC hydrochloride. As shown in **Figure 4D**, transfer of DKI monocytes induced an earlier onset and more severe colitis in WT mice (**Figure 5H**); however, the inhibition of arginase I activity ameliorated the severity of colitis conferred by the DKI monocyte transfer (**Figure 5H**). These findings demonstrate that the severe colitis phenotype observed in the DKI mice was the result of increased arginase I activity in STAT5 tetramer-deficient MDCs.

### Overexpression of arginase I in DKI monocytes limits the production of protective nitric oxide

**Figure 6:**
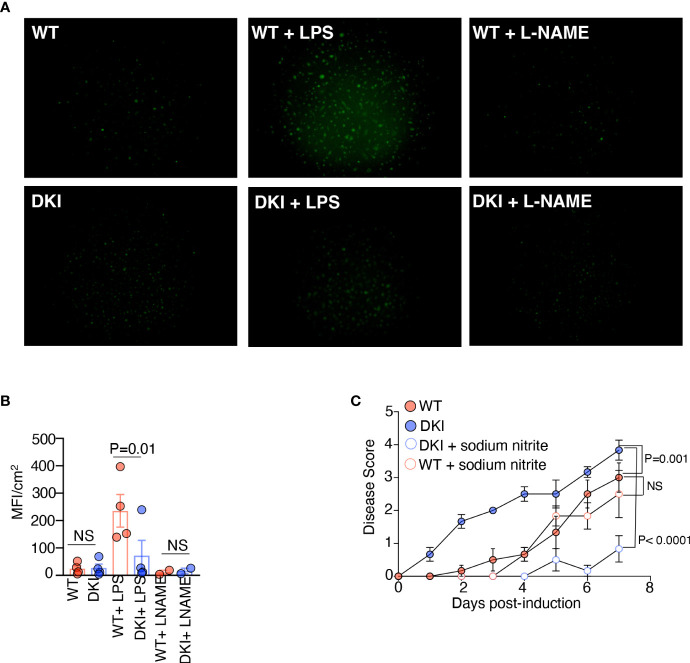
Overexpression of Arginase I in DKI Monocytes Limits the Production of Protective Nitric Oxide. **(A, B)** WT and DKI bone marrow monocytes were differentiated with GM-CSF for 8 d. Some MDCs were stimulated with either LPS or L-NAME for 16h. Cells were stained with DAF-FM diacetate for 30 min. **(A)** Representative images and **(B)** Quantification of data generated from 3 independent experiments. **(C)** WT and DKI mice were given drinking water containing 1% DSS or 1% DSS with 1 mM sodium nitrite for 7 days. Disease scores are shown. Data are from 2 independent experiments with n=6. **(B, C)** Data shown are mean ± SEM. Statistical significance is indicated by the P-value with NS meaning no statistical significance. **(B)** Paired *t* test. **(C)** Area under the curve with one way ANOVA and Tukey’s test for multiple comparisons.

Finally, we sought to identify the mechanism by which increased arginase I activity in monocytes promotes colitis. In monocytes and MDCs, arginine is metabolized by arginase I and iNOS. Arginase I hydrolyzes arginine to ornithine, which is further converted into proline and polyamines, whereas iNOS converts arginine into citrulline and nitric oxide (NO) (33). In addition, arginase I is known to inhibit the activity of iNOS *via* multiple mechanisms (34, 35). Previous studies have shown that dietary supplementation with L-arginine confers protection against DSS-induced colitis; however, dietary supplementation with L-ornithine and L-proline does not provide protection (36). In contrast, the protective effect of L-arginine is eliminated in the absence of iNOS (37). These data led us to speculate that the increase in arginase I expression in the DKI monocytes inhibited the production of protective NO, thereby exacerbating colitis severity. We first compared the levels of NO in WT and DKI MDCs following LPS stimulation. The WT MDCs produced more NO compared to the DKI MDCs following LPS stimulation, and as expected, the production of NO was inhibited by the NOS inhibitor, L-NAME (**Figures 6A, B**). Given that the expression of iNOS in WT and DKI MDCs was comparable (**Figures 5C, D**), the reduction of NO production in the DKI monocytes was possibly due to increased arginase I activity and subsequent L-arginine consumption. Next, colitis was induced in WT and DKI mice supplemented with sodium nitrite, which provides a sustained concentration of NO. Interestingly, the addition of sodium nitrite did not impact the colitis severity in WT mice, but significantly ameliorated colitis in DKI mice (**Figure 6C**). These results suggest that STAT5 tetramer deficiency may lead to the reduction of protective NO which can be modulated by sodium nitrite supplements. However, the addition of exogenous source of NO more than physiological concentration does not have further beneficial effect. Taken together, these data suggest that the increase in arginase I activity in the DKI monocytes promotes colitis severity potentially *via* the regulation of protective NO synthesis.

## Discussion

In this study, we have established that STAT5 tetramers play an essential role in regulating monocyte fate and DSS-mediated colonic pathogenesis. We determined that GM-CSF-induced STAT5 tetramerization in monocytes promotes their differentiation into a functionally and genetically distinct population of moDCs. However, in the absence of STAT5 tetramers, dysregulated GM-CSF signaling promotes a macrophage phenotype. Previous studies have identified distinct monocyte progenitor populations that give rise to moDCs and monocytes-derived macrophages (25, 38). These progenitors were thought to account for the heterogeneity of the monocyte-derived cell population. However, we determined that GM-CSF-mediated STAT5 tetramers control the fate of monocyte differentiation, despite there being similar proportions of these progenitor populations in the bone marrow of the WT and DKI mice. Thus, we propose that STAT5 tetramers can regulate an intrinsic program that regulates monocyte differentiation. Interestingly, a previous study demonstrated that in response to GM-CSF, IRF4-deficient monocytes differentiate into macrophages but not moDCs (39). The morphology and the expression of macrophage-associated cell surface markers bear resemblance to the DKI monocytes that have been differentiated with GM-CSF. Additionally, we determined that the expression of *Ifr4* was regulated by GM-CSF-mediated STAT5 tetramers. Therefore, it is possible that IRF4 acts downstream of STAT5 tetramers to regulate monocyte differentiation. This warrants further investigation.

Using the DSS model of colitis, we established that STAT5 tetramers in monocytes play a critical role in conferring protection against intestinal inflammation. GM-CSF is known to ameliorate colitis severity (40–43). In fact, there have been several clinical trials that have sought to treat IBD patients with recombinant GM-CSF (44), and while not efficacious in all patients, treatment with recombinant GM-CSF was able to successfully decrease colitis severity (13–16). However, the mechanism though which GM-CSF ameliorates colitis has not been fully elucidated. We determined that the inhibition of GM-CSF was able to ameliorate the severe colitis observed in the DKI mice. We propose that GM-CSF acts on monocytes to promote STAT5 tetramerization. When STAT5 tetramerization is inhibited, dysregulated GM-CSF signaling renders the monocytes pathogenic. It is important to note that unlike the known protective role of GM-CSF, there is evidence to support both a pathogenic and protective role of monocytes in colitis (45–47). Previous studies have demonstrated that DSS promotes the recruitment of inflammatory monocytes to the colon, where they can differentiate into monocyte-derived macrophages and dendritic cells. These cells can respond to gut-resident bacterial *via* TLR signaling and thus promote intestinal inflammation and associated tissue damage (46, 47). However, the ablation of macrophages and dendritic cells was found to exacerbate epithelial cell injury and inflammation following colitis induction (30), suggesting that monocytes may also have an immunomodulatory role in colitis. This led us to investigate whether monocyte recruitment protected against or exacerbated colitis severity. Using the *Ccr2* ^RFP/RFP^ mice strain, we determined that monocytes are required to protect against severe colitis. Moreover, we demonstrated that the transfer of WT monocytes to DKI mice was able to ameliorate colitis severity. These findings suggest that GM-CSF-mediated STAT5 tetramers are critical for promoting an immunomodulatory monocyte phenotype in the context of intestinal inflammation.

The transfer of DKI monocytes into WT recipient mice significantly increased colitis severity. Consequently, we sought to determine the mechanism through which the inhibition of STAT5 tetramerization in monocytes promoted their pathogenicity. We determined that the expression and activity of arginase I was significantly increased in DKI monocytes, and the expression was increased in the colons of the DKI mice during the early phase of colitis. Additionally, inhibiting arginase I activity in the DKI monocytes was able to decrease colitis severity. In line with these observations, studies have suggested that arginase I promotes colitis pathogenesis (31, 32). Importantly, we demonstrated that a sustained concentration of NO decreased colitis severity in DKI mice. In monocytes and MDCs, L-arginine is competitively metabolized by arginase I and iNOS (33). We propose that the high levels of arginase I in the DKI monocytes consume the available L-arginine, thereby limiting the synthesis of NO through the reduction of iNOS activity. There are several studies that have demonstrated that low levels of NO can limit intestinal injury, leukocyte infiltration, and proinflammatory cytokine production in murine models of colitis (48–50). Though the precise mechanism has not been fully elucidated, there is evidence to suggest that NO may act as an antioxidant that has the potential to limit superoxide-induced tissue damage by inhibiting superoxide release or by scavenging superoxide (51, 52), which may limit intestinal inflammation and subsequent immune cell recruitment. It is also possible that the increase in arginase I production and activity by the DKI monocytes and thus polyamine synthesis may increase cell proliferation. This could result in pathogenic colonic hyperplasia, which has been correlated with increased colitis severity (53). However, the pathological assessment of our WT and DKI colons demonstrated that there was minimal hyperplasia present following both 4 and 7 days of treatment with DSS. Additionally, dietary supplementation with L-ornithine, which is metabolized to polyamines, does not provide protection against colitis (36). Therefore, we propose that the increased production of arginase I following dysregulated GM-CSF signaling in the DKI monocytes limits the synthesis of protective NO.

This study has established a critical role of GM-CSF-mediated STAT5 tetramers in conferring protection against colitis *via* suppression of arginase I, and in turn reduces the production of protective NO. The pathological significance of STAT5 tetramers in regulating differentiation of moDC versus macrophages needs to be further investigated. We previously demonstrated that, in stark contrast to their role in colitis, STAT5 tetramers promote autoimmune-mediated neuroinflammation. We determined that STAT5 tetramers regulated the expression of CCL17 in MDCs, which promoted the migration and pathogenicity of Th17 cells in the spinal cord meninges (21). The STAT5 tetramer-dependent regulation of monocyte function appears to be highly context specific, and the differences, in this case, seem to be largely driven by the interplay between monocytes and other immune and non-immune cells that are also involved in promoting or restricting inflammation. For instance, in colitis, colonic epithelial cells are thought to be the major cellular source of GM-CSF, which acts on the monocytes to confer protection (44). However, in neuroinflammation, pathogenic Th17 cells are the major cellular source of GM-CSF, which acts on the MDCs to promote their pathogenicity (54). The findings from these studies highlight the opposing roles of STAT5 tetramers in governing inflammation. It is important that these opposing roles be considered related to future therapeutic interventions that seek to target downstream mediators of the GM-CSF-STAT5 tetramer signaling pathway for the treatment of chronic inflammatory conditions.

## Methods

### Mice

The generation of the *Stat5a-Stat5b* double knockin (DKI) N-domain mutant mice was described previously (19). The DKI mice are on a C57BL/6J (B6) background. *Ccr2* ^RFP/RFP^ and *Ccr2* ^+/RFP^ mice (JAX 017586) and OT-II transgenic mice (JAX 004194) mice were purchased from The Jackson Laboratories. Mice were bred and housed under specific-pathogen-free facilities at West Virginia University Health Sciences Center. All studies were conducted in accordance with West Virginia University Animal Care and Use Committee guidelines.

### Monocyte isolation and differentiation *in vitro*

Bone marrow was harvested from WT and DKI mice as previously described (55). The epiphyses from both the tibias and femurs were removed. A total of 5 mL RPMI medium was passed through the bones using a 26G needle. Monocytes were enriched by negative selection using Monocyte Isolation Kit (Miltenyi Biotec 130-100-629) and LS columns (Miltenyi Biotec 130-042-401) or using EasySep Mouse Monocyte Isolation Kit (Stemcell Technologies 19861). The enriched monocytes were resuspended in RPMI and were seeded in a 24-well plate at a density of 5 x 10^5^ cells/mL. Cells were kept at 37*°*C for 1 h, then the RPMI was replaced with 1 mL of complete RPMI containing 10% fetal bovine serum (Cytiva SH30071.03HI), 2mM L-glutamine (Gibco 25030-08), 0.1 mg/mL penicillin/streptomycin (Gibco 15240-062), and 55 μM β-mercaptoethanol (Gibco 21985-023) with 2, 5, or 20 ng/mL GM-CSF (Peprotech 315-03). On days 3 and 6, the cells were supplemented with 500 μL complete RPMI containing 2, 5, or 20 ng/mL GM-CSF. For functional assays, differentiated cells were harvested on day 8. For RNA-Seq and flow cytometric analysis, differentiated cells were harvested on days 3, 6 and 9. For the detection of iNOS and arginase I, arginase I activity, and nitric oxide, the GM-CSF differentiated cells were stimulated with 100 ng/mL LPS (O111:B4, Sigma-Aldrich) for 16 h, prior to harvesting. In some experiments, monocytes were differentiated in the presence of M-CSF (Peprotech 315-02) as described above or directly stimulated with 100 ng/mL LPS for 16 h without prior GM-CSF stimulation. To harvest cells, medium was collected, and cells were washed once with PBS. Cells were detached from the plate by incubating with 500 μL accutase (Innovative Cell Technologies AT 104-500) for 5 min at 37*°*C. The suspended cells were collected. The wells were then gently scraped to collect any remaining cells.

### Single-cell isolation from the lungs, spleens, and peritoneal cavity

To obtain cells from the lungs, mice were deeply anesthetized using ketamine/xylazine combination. Whole-body perfusion was performed by administering 20 mL of cold PBS *via* the left cardiac ventricle. Lungs were excised and cut into pieces 1-2 mm in size and placed into 5 mL of digestion buffer containing collagenase D (1 mg/mL) and DNase I (200 µg/mL) in HBSS. The tissues were vortexed and incubated in a 37°C water bath for 45 min with vigorous vortexing every 8-10 min. Digests were pushed through 100-µm cell strainers and contents were centrifuged for 10 min at 380 x g. Supernatant was discarded and tissue digests were resuspended in PBS with 1% FBS. To obtain cells from the spleens, spleens from euthanized mice were homogenized using syringe plunger and then pushed through 100-µm cell strainers. Red blood cells were lysed using ACK lysing buffer. The cells were then washed and resuspended in PBS with 1% FBS. To obtain cells from the peritoneal cavity, euthanized mice were injected intraperitoneally with 5 mL cold PBS. The peritoneal cavity was gently massaged, the injected PBS containing cells was then collected.

### Antibody staining and flow cytometric analysis

Differentiated monocytes were harvested as described above on days 3, 6, and 9. The single cell suspensions were incubated with anti-CD16/32 (Biolegend 93) at a 1:100 dilution on ice for 10 min and cells were then stained with the following conjugated antibodies (antibodies used in this study were purchased from BioLegend if not otherwise indicated) at 4°C for 20 min: CD11c-Percp/Cy5.5 (N418), MHCII-BV510 (M5/114.15.2), F4/80-BV421 (BM8), CD115-APC (AFS98), CD80-PE (16-10A1), CD86-APC (GL-1), CD11b-FITC (N418), Ly6C-PE (HK1.4), CCR2-BV421 (SA203G11), MerTK-PE (eBiosciences DSSMMER), GM-CSFRα-PE (R&D Systems FAB6130P), and GM-CSFRβ-PE (BD Biosciences JORO50). Dead cells were excluded by using LIVE/DEAD Fixable Near-IR Dead Cell Stain Kit (ThermoFisher L34976). The cells were washed and resuspended in staining buffer (1% FBS in PBS), then analyzed on a BD LSRFortessa flow cytometer. For the intracellular staining of arginase I and iNOS, the cells were surface stained, then fixed and permeabilized using Fixation/Permeabilization Solution Kit (BD Biosciences 555028) according to the manufacturer’s recommendations. Following permeabilization, the cells were stained with arginase I-APC (AlexF5) and iNOS-PE (CXNFT). The cells were washed in permeabilization buffer, then were resuspended in staining buffer prior to analysis. For experiments that characterized bone marrow progenitor populations (25), bone marrow cells were incubated with anti-CD16/32 at a 1:100 dilution on ice for 10 min and cells were then stained with the following conjugated antibodies: Ly6C-PE (HK1.4), Flt3-Percp/eFluor (A2F10), CD11c-PE/Cy7 (N418), CD115-APC (AFS98), SIPRα-APC/Cy7 (P84), MHC II-BV421 (M5/114/15/2), Ly6G-BV510 (1A8), and TCRβ−BV510 (H57-597). Cells were then fixed and permeabilized using Fixation/Permeabilization Solution Kit (Ebioscience 00-5523) according to the manufacturer’s recommendations. Following permeabilization, the cells were stained with PU.1-AF488 (7C2C34). The cells were washed in permeabilization buffer and then resuspended in staining buffer prior to analysis.

### Detection of STAT5 phosphorylation

Monocytes were enriched from the bone marrow of WT and DKI mice as described above. Monocytes were seeded at a density of 1 x 10^5^ cells/well in a 96-well plate in complete medium and were rested at 37°C for 30 min. The monocytes were stained with CD11b-FITC (N418), Ly6C-PE (HK1.4), and LIVE/DEAD Fixable Near-IR Dead Cell Stain Kit (ThermoFisher L34976) for 20 min at 37°C. Following surface staining, the monocytes were stimulated with 100 ng/mL GM-CSF (Peprotech 315-03) for 15 min at 37°C. The stimulated monocytes were then fixed in 2% paraformaldehyde in PBS at 37°C for 10 min. Following fixation, the monocytes were permeabilized with 90% ice cold methanol in PBS and were incubated at -20°C for 30 min. Monocytes were washed with staining buffer, then stained with pSTAT5-PE (BD Biosciences 47/Stat5 (pY694)) at room temperature for 30 min. The monocytes were washed and resuspended in staining buffer. Cells were analyzed on a BD LSRFortessa.

### Assessment of cell morphology

Monocytes were enriched and differentiated as described above. After 8 days of differentiation, the cells were cytospun onto a glass slide, then stained with the Wright Giemsa staining kit and imaged using the MFI Zeiss Tissue Culture Microscope.

### Phagocytosis assay

Monocytes were enriched and differentiated with GM-CSF as described above. Following 8 days of differentiation, the cells were harvested and resuspended in complete medium to a concentration of 2 x 10^5^ cells/100 μL. Cells were then incubated with 200 μg/mL AF488 conjugated ovalbumin (Invitrogen 034781) at either 37°C or 4°C for 1 h. Cold PBS was added to the cells, which were then washed 2 times with PBS. Cells were then stained with CD11c-Percp/Cy5.5 (N418), MHCII-BV510 (M5/114.15.2), and LIVE/DEAD Fixable Near-IR Dead Cell Stain Kit (ThermoFisher L34976), as described above. Cells were analyzed on a BD LSRFortessa.

### Antigen presentation assay

Inguinal, axillary, brachial lymph nodes, and spleens were harvested from OT-II transgenic mice. Naïve CD4^+^ T cells were enriched using Naïve CD4^+^ T cell Isolation Kit. (Miltenyi 130-104-453 or Stemcell Technologies 19765). The naïve T cells were then labeled using the CellTrace Violet Cell Proliferation Kit (ThermoFisher C34571) according to the manufacturer’s recommendations. The labeled T cells were seeded in a 96 well plate at a density of 5 x 10^4^ cells/200 μL complete medium. Monocytes from WT and DKI mice were enriched and differentiated as described above. Following 8 days of differentiation, the cells were added to the wells containing naïve CD4^+^ T cells in the indicated ratios. The cells were then stimulated with 2, 5, or 20 μg/mL ovalbumin peptide (OVA_323-339_ AnaSpec AS-27025) or 2 μg/mL ovalbumin protein (InvivoGen vac-pova). The cells were co-cultured for 72 h at 37°C. Following 72 h of co-culture, the T cells were harvested and stained with LIVE/DEAD Fixable Near-IR Dead Cell Stain Kit (ThermoFisher L34976) and CD4-APC (RM4-5) as described above. T cells proliferation was analyzed on a BD LSRFortessa.

### Oxidative burst assay

Monocytes were enriched and differentiated with GM-CSF as described above. Following 8 days of differentiation, the cells were stimulated with 100 ng/mL LPS and incubated wit 500 μL nitro blue tetrazolium chloride (ThermoFisher N6495) for 15 min at 37°C. The colorimetric change that signified an oxidative burst was imaged and quantified on the EVOS Cell Imaging System (ThermoFisher).

### Cytokine and chemokine bead array

Monocytes were enriched and differentiated as described above. Following 8 days of differentiation, 500 μl of cultured supernatants were collected and centrifuged at 15,870 x g for 3 min. Supernatants were collected and stored at -80°C until analysis. A chemokine (Biolegend 740451) bead arrays were performed according to the manufacturer’s protocol and analyzed on a BD LSRFortessa.

### RNA-Seq library preparation and sequencing

Monocytes were enriched and differentiated as described above and then harvested on day 0, 3, or 6. RNA was extracted using the Zymo RNA miniprep kit (Zymo Research), and 500 ng RNA was used for RNA-Seq library preparation with the Kapa mRNA HyperPrep Kit (Kapa Biosystems KK8580) and indexed with NEXTflex DNA Barcodes-24. After the final amplification, samples were loaded onto 2% E-Gel pre-cast gels (ThermoFisher), and 250 to 400 bp DNA fragments were recovered and purified with Zymoclean Gel DNA Recovery Kit (ThermoFisher). Barcoded samples were mixed and sequenced on an Illumina HiSeq2000 system.

### RNA-seq data analysis

RNA-Seq data processing followed our previous procedures (56, 57). In brief, single end RNA-Seq reads were aligned to the mouse reference genome (mm10) by sub-read (58). The number of reads mapped to transcripts annotated by RefSeq at gene level was counted by FeatureCounts (59). Gene expression level was quantified by RPKM (60). Differentially expressed genes were predicted by EdgeR with the following thresholds: fold-change > 1.5, FDR < 0.05, count per million (CPM; log_2_) > 0, and expression level in at least one condition (RPKM) > 2 (61).

### DSS-induced colitis

Colitis was induced by incorporating DSS into the drinking water as previously described (62). Male WT and DKI mice or *Ccr2* ^+/RFP^ and *Ccr2* ^RFP/RFP^ mice between 12-16 weeks of age were randomly assigned to treatment groups for each experiment. One percent colitis grade DSS (MP Biomedicals 160110) was dissolved into autoclaved drinking water. Mice were restricted to only the DSS-treated water for 7 days. The weights and colitis scores of the mice were measured daily. Colitis score on a five-point scale was utilized: 0: Normal stool; 1: Water retention in the stool, softening of the stool; 2: Presence of occult blood measured by hemoccult test (Beckman Coulter 61130); 3: Presence of visible trace blood in the stool; 4: Presence of acute rectal bleeding; 5: Presence of gross rectal bleeding. Data from all mice were included without exclusion. Mice were scored in an unblinded manner. To neutralize GM-CSF activity, 300 μg of anti-GM-CSF antibody (Bioxcell MP1-22E9) or rat IgG2a isotype control antibody against trinitrophenol (Bioxcell 2A3) diluted in 200 μl PBS was administered to the recipient mice *via* intraperitoneal injections on days 0, 2, and 4 following colitis induction. To inhibit arginase I activity, mice were intraperitonially injected with 600 μg/200 μL BEC hydrochloride (Sigma SML1384) on days 0, 1, and 2 following colitis induction. To supplement nitrite, 1 mM sodium nitrite (Sigma Aldrich 077-010-00-4) was added to the DSS-treated water, as previously described (63). Colitis scores were evaluated for 7 days.

### DSS-induced colitis with monocyte transfer

Monocytes were enriched from the bone marrow of WT and DKI mice as described above. The enriched monocytes were resuspended to 1 x 10^6^ cells/200 μL PBS and were transferred to the indicated recipient mice *via* intraperitoneal injections on days 0 and 2 following colitis induction. In some experiments, BEC hydrochloride (600 μg/200 μL) was administered on days 0, 1, and 2 *via* intraperitoneal injections. Colitis scores were evaluated for 7 days.

### Arginase I activity assay

WT and DKI monocytes were enriched and differentiated with GM-CSF as described above. Some cells were stimulated with 100 ng/mL LPS (O111:B4, Sigma-Aldrich) for 16 h prior to harvesting. After 8 d of differentiation, the activity of arginase I determined using the Arginase Activity Assay Kit, according to manufacturer’s protocol (Abcam ab180877). A total of 5 μL of lysate was used for each reaction. Reaction time was 20 min.

### DAF-FM diacetate staining

WT and DKI monocytes were enriched and differentiated with GM-CSF as described above. Some cells were stimulated with 100 ng/mL LPS (O111:B4, Sigma-Aldrich) or 100 μM L-NAME (R&D Systems 0665/100) for 16 h prior to harvesting. After 8 d of differentiation, medium was removed and the wells were washed with warm Dulbecco’s phosphate buffered saline (DPBS, ThermoFisher 14040141). The cells were stained with 1 μM DAF-FM Diacetate (ThermoFisher D238440) in DPBS for 30 min at 37°C. The cells were washed with warm DPBS then imaged on the Zeiss Fluorescent Microscope.

### Pathological assessment of the colons

The colons were harvested from the mice at 4 or 7 days following colitis induction. The colons were prepared using Swiss roll technique as previously described (64), and fixed in 4% paraformaldehyde in PBS for 24 h at 4°C. Following fixation, the colons were washed with PBS and were placed in 30% sucrose in PBS overnight at 4*°*C two times. The colons were then embedded in O.T.C. compound (Fisher Health Care 4585). and cut into 12-μm sections on a cryostat (ThermoFisher HM525 NX). The sections were stained with a standard H&E staining protocol and were scored in a single-blinded manner by a pathologist. The extent of inflammation and erosion/ulceration were examined as the percent of the colon that was affected. The following scale was utilized: 0 = 0%, 1 <5% affected, 2 = 5% to 25% affected, 3 = 25% to 50% affected, 4 = >50% affected. The extent of epithelial hyperplasia present was determined by examining the entire colons section. The following scoring system was utilized: 1: focal, 2: multifocal/regional, and 3: extensive.

### Immunofluorescence staining

The colons were harvested from the mice at 4 d following colitis induction. The colons were prepared using Swiss roll technique as previously described (64), and fixed in 4% paraformaldehyde in PBS for 24 h at 4*°C.* Following fixation, the colons were washed with PBS and were placed in 30% sucrose in PBS overnight at *4*°C two times. The colons were then embedded in O.T.C. compound (Fisher Health Care 4585) and cut into 12-μm sections on a cryostat (ThermoFisher HM525 NX). The slides containing 12-μm frozen colon sections were treated with acetone for 5 min at -20°C. After drying, the slides were incubated with tris-buffered saline with 0.1% Tween 20 (TBS-T) for 15 min and further washed 2 times with TBS-T, followed by incubating with 1% Triton X-100 diluted in TBS-T for 30 min at room temperature. The slides were then blocked with 2% goat serum for 2 h at room temperature, washed 6 times with TBS-T, and stained with anti-arginase I antibody (Cell Signaling Technologies D4E3M, 1:100) in 2% goat serum overnight at 4°*C*. Slides were washed 3 times with TBS-T, then stained with FITC-conjugated goat anti-rabbit secondary antibody (Invitrogen A11008, 1:1000) in 2% goat serum for 2 h at room temperature. The slides were washed 3 times with TBS-T, then stained with DAPI for 3 min at room temperature. Slides were washed 3 times with TBS-T, then were cover slipped with antifade mounting medium (Vector Laboratories H-1000). For colons samples from *Ccr2* ^+/RFP^ mice, the samples were prepared and fixed as described above. The slides were then washed 7 times with PBS, stained with DAPI for 10 min, then washed 3 times with PBS. Slides were cover slipped with antifade mounting medium (Vector Laboratories H-1000).

### Image collection and analysis

Large stitch tiled images from anti-arginase I antibody-stained sections were collected on the Nikon A1R confocal with N-SIM E microscope with the PlanFluor 40x/0.75 objective. Images were generated using NIS Elements software. The number of arginase I expressing cells was determined using General Analysis. The 488 channel (arginase I) was subjected to auto contrasting and thresholding. The number of objects was determined and normalized to the area with signal (percent of area with signal multiplied by object count), Analysis was performed on 3 z-stack images per colon. Images from H&E-stained slides and slides obtained from treated *Ccr2* ^+/RFP^ and *Ccr2* ^RFP/RFP^ mice were collected on the MIF Olympus Slide Scanner using the 20x objective. Images from the DAF-FM diacetate-stained MDCs were collected on the Zeiss Fluorescent Microscope using the 10x objective. The number of NO-producing cells was calculated using ImageJ. The fluorescent intensity was normalized to the area (cm^2^).

### Statistical analysis

Statistical analysis was conducted using GraphPad Prism. The severity of colitis between experimental groups was compared by determining the area under the curve with p-values being determined using two-tailed Student’s t test or one-way ANOVA with Tukey’s *post hoc* analysis for multiple comparisons. Significance was defined as p-values < 0.05. Statistical test, extant n values, and the number of each experimental replicate are denoted in the Figure Legends.

## Data availability statement

The datasets presented in this study can be found in online repositories. The names of the repository/repositories and accession number(s) can be found below: GSE207676 (GEO).

## Ethics statement

The animal study was reviewed and approved by West Virginia University Animal Care and Use Committee.

## Author contributions

KM and EW designed research; KM, WZ, EW performed research; J-XL contributed new reagents/analytic tools; KM, HA, LW, AA, PL, GH, EW analyzed data; KM, GH, WL, EW wrote the paper. All authors contributed to the article and approved the submitted version.
