# Supplementary figures and images for: Tetramerization of STAT5 regulates monocyte differentiation and the dextran sulfate sodium-induced colitis in mice
Source: Front Immunol. 2023 Apr 20;14:1117828. doi: 10.3389/fimmu.2023.1117828 (PMC10157487; doi:10.3389/fimmu.2023.1117828)

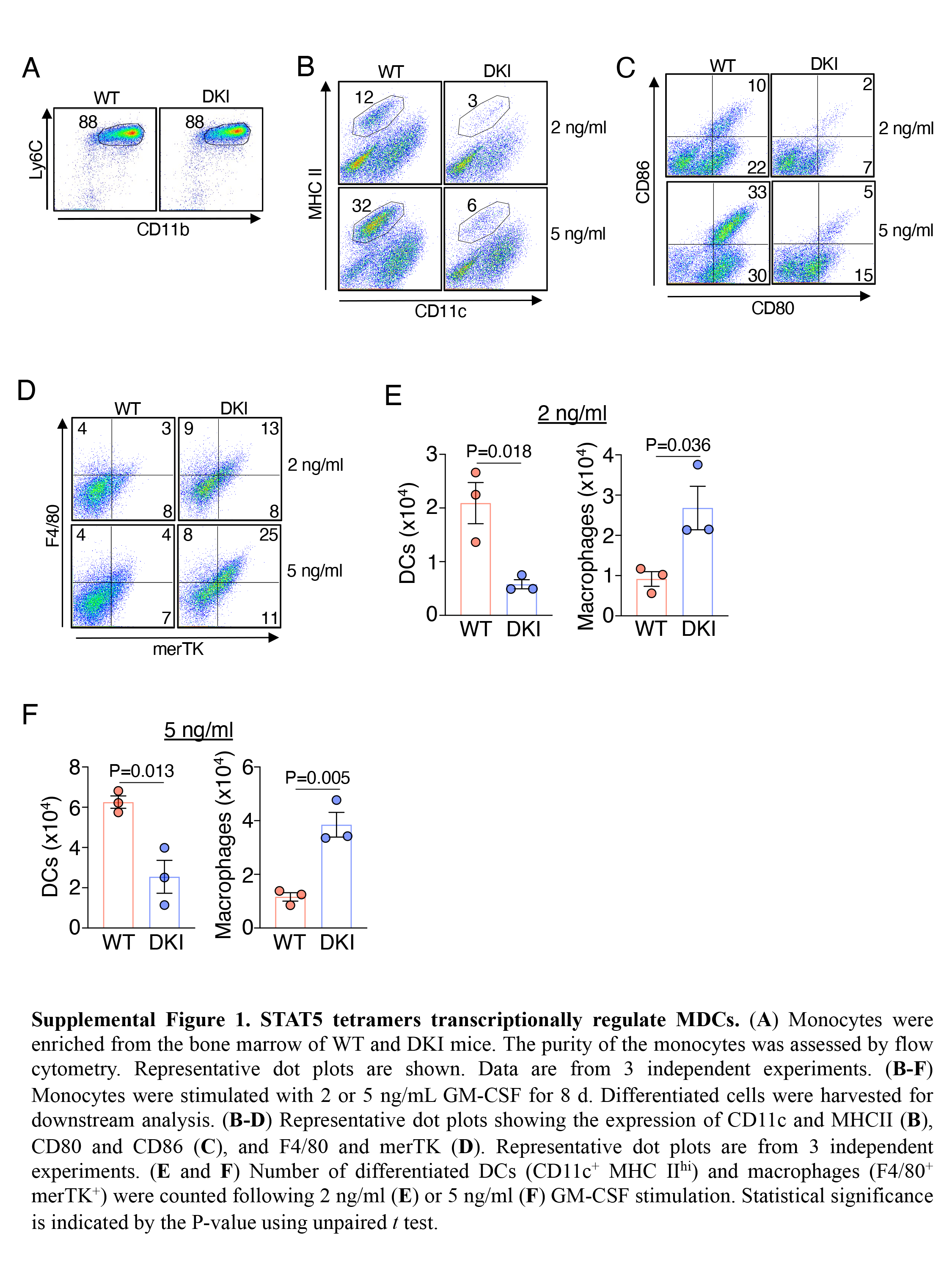

Supplement: Supplementary file 1 [file Image_1.tif]

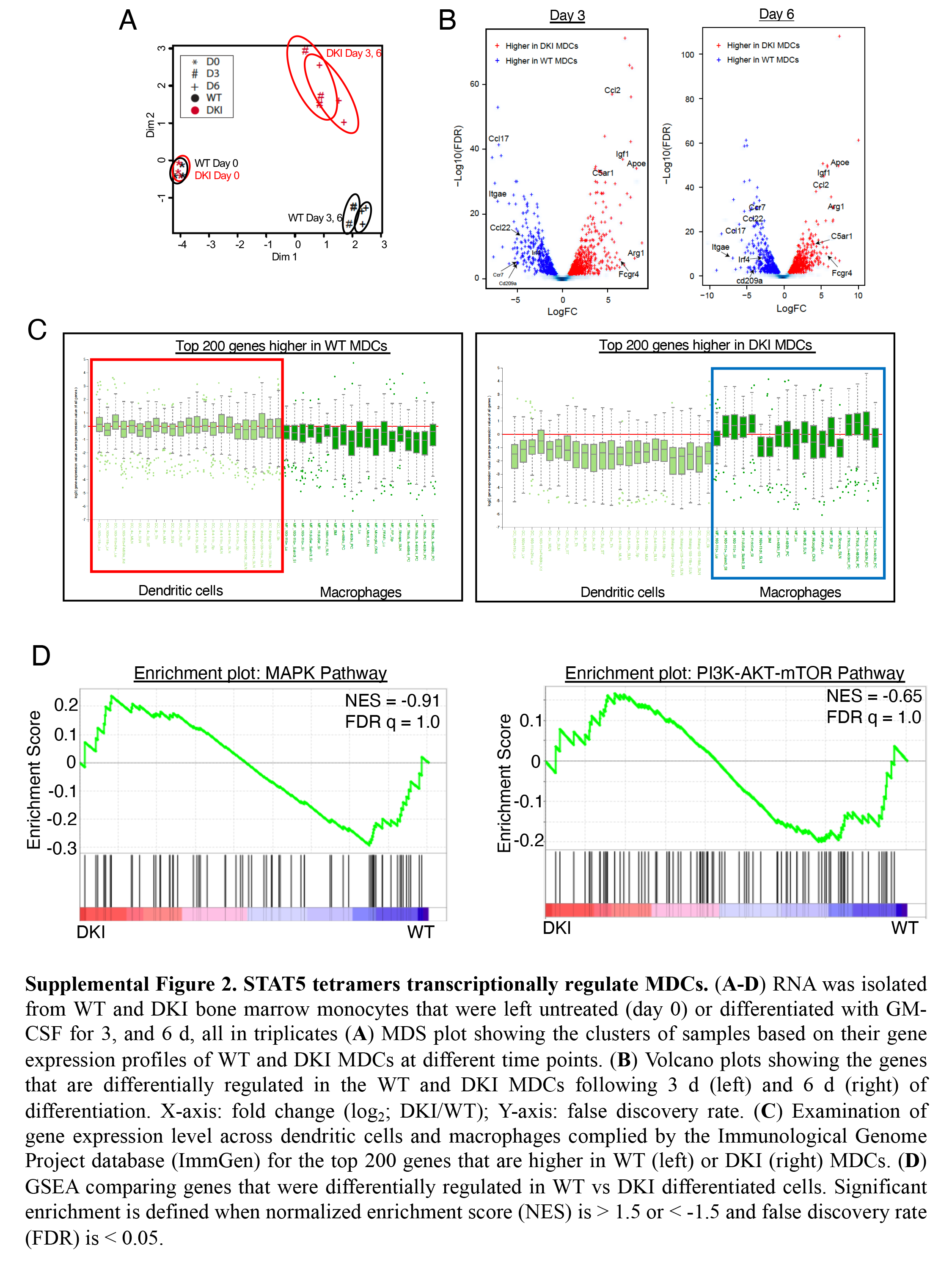

Supplement: Supplementary file 2 [file Image_2.tif]

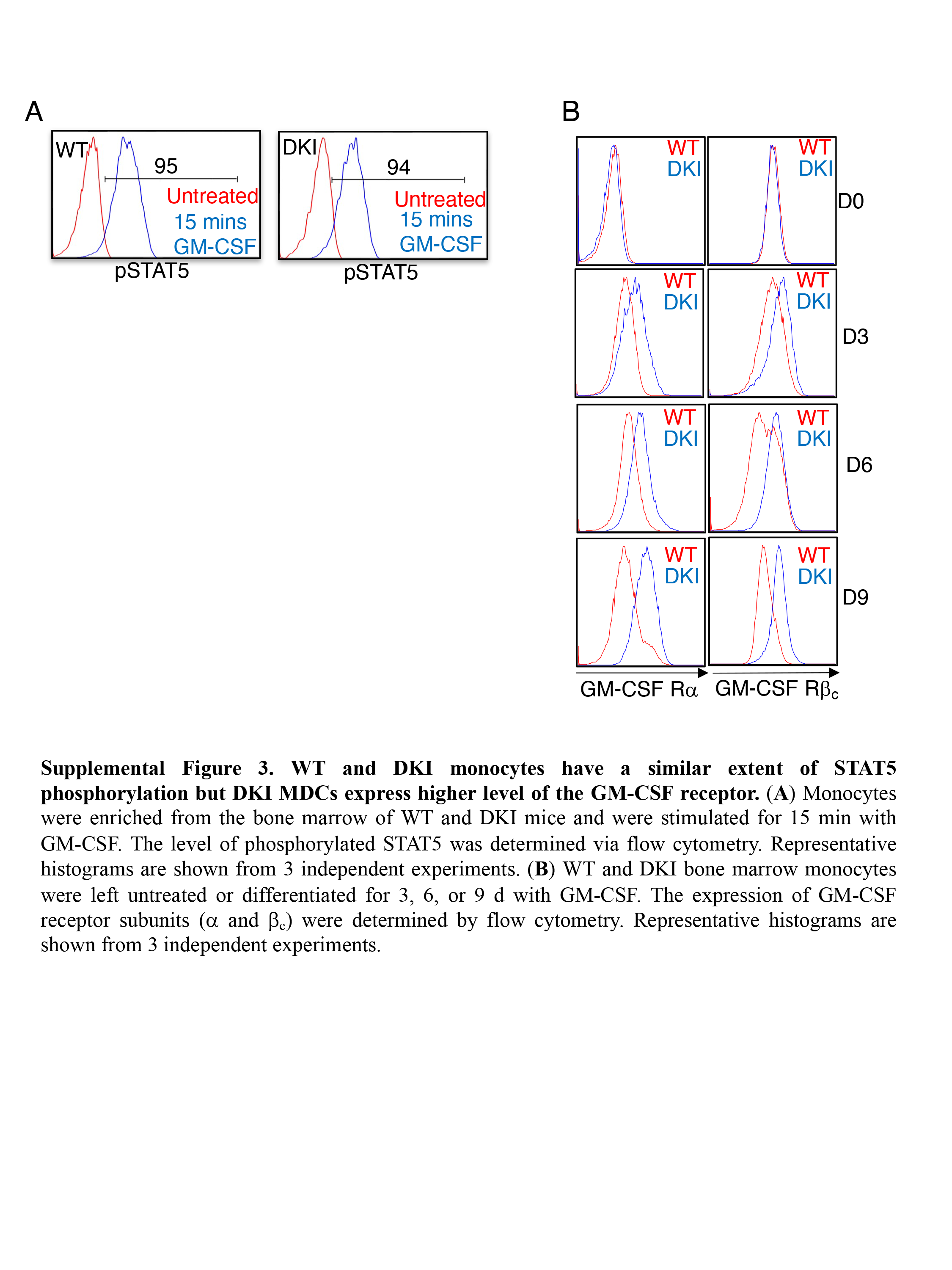

Supplement: Supplementary file 3 [file Image_3.tif]

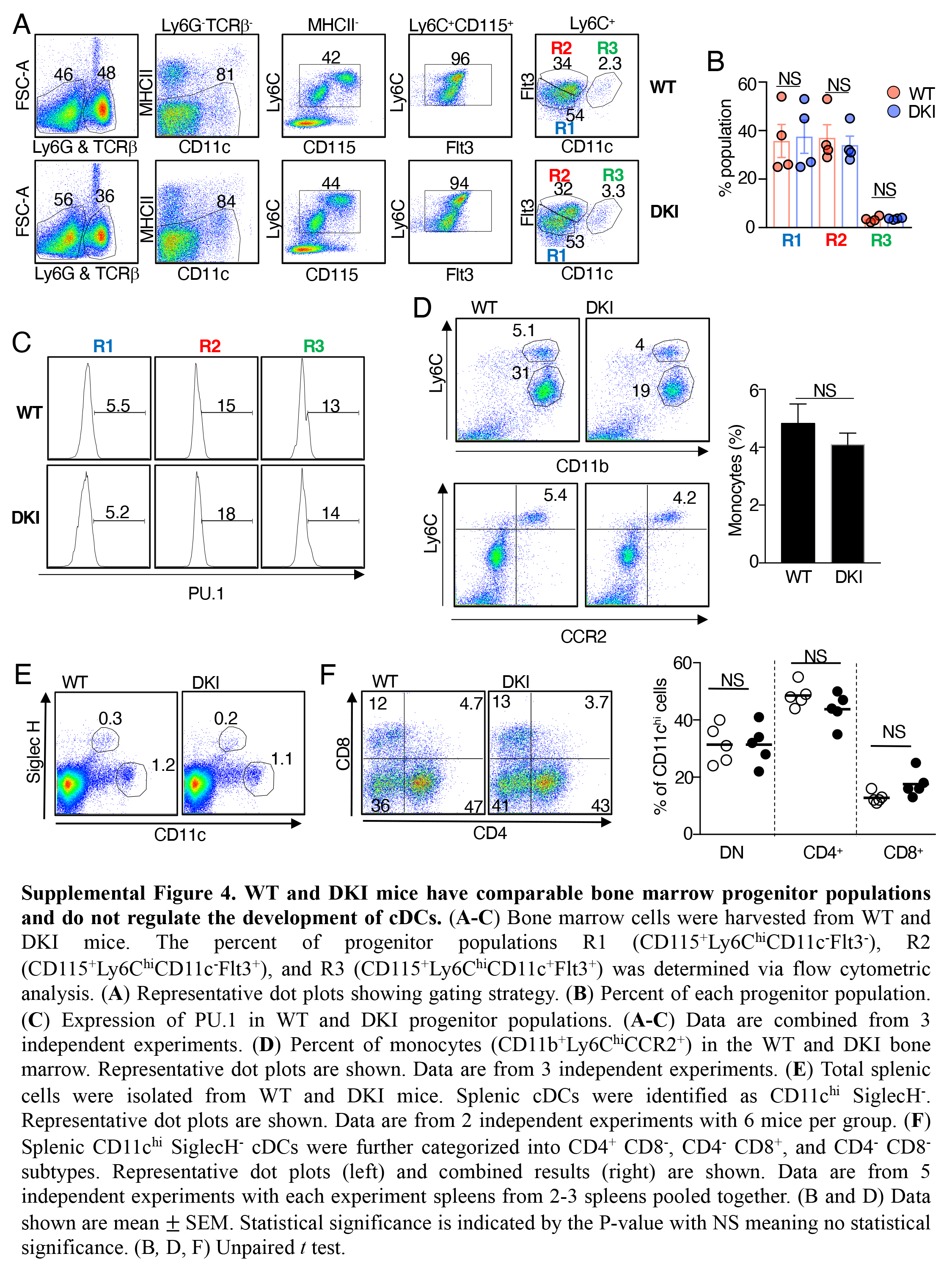

Supplement: Supplementary file 4 [file Image_4.tif]

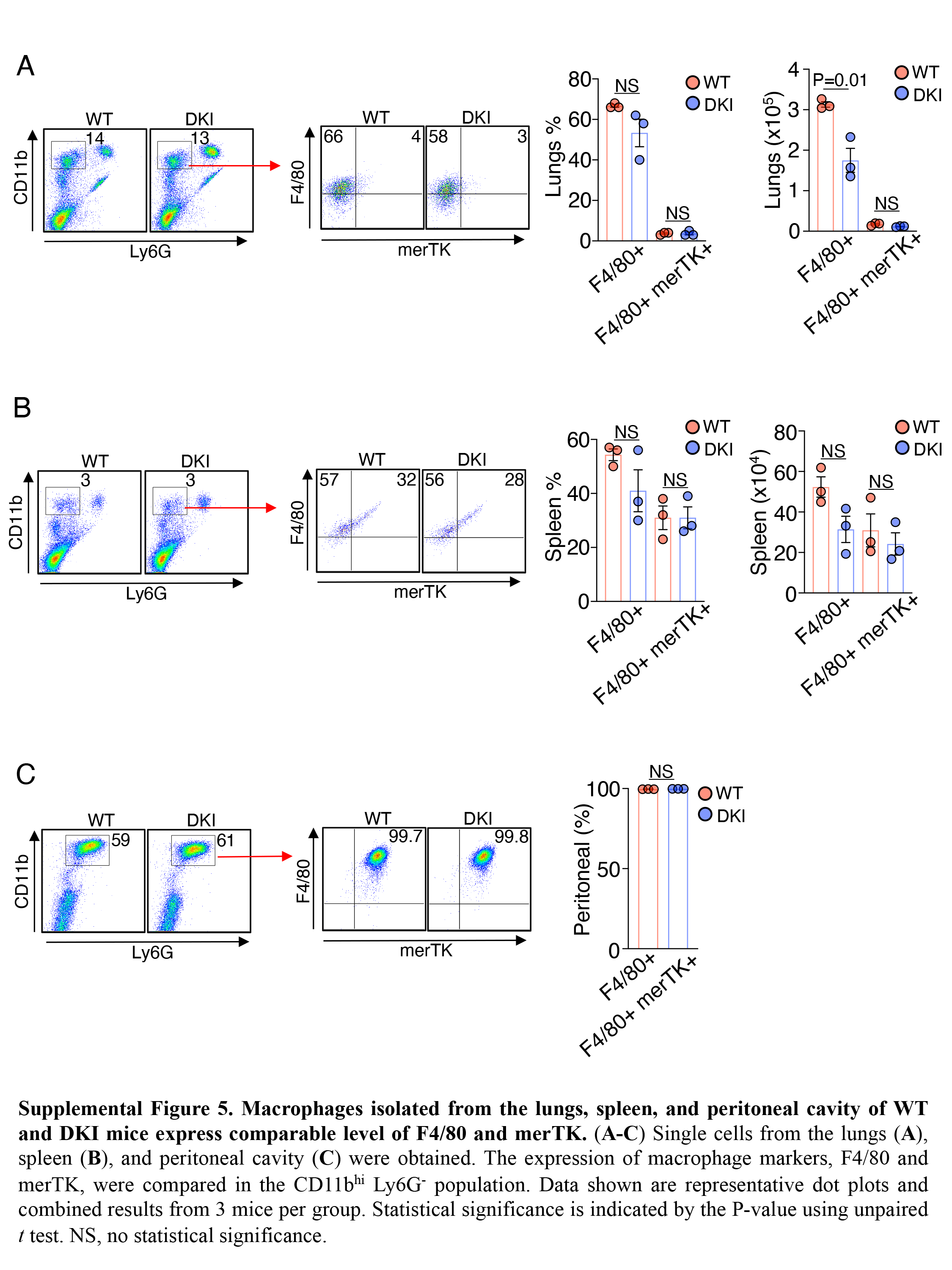

Supplement: Supplementary file 5 [file Image_5.tif]

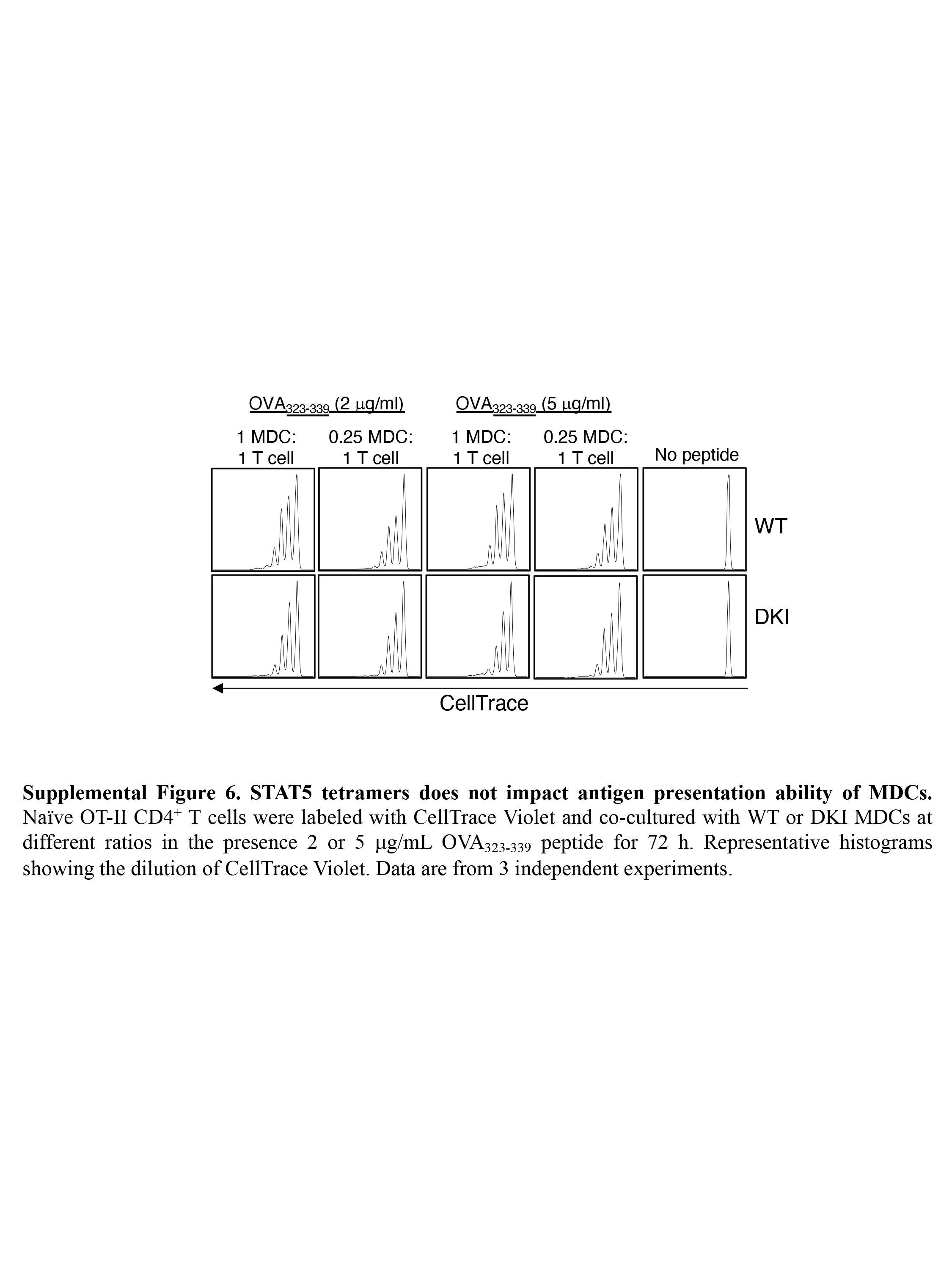

Supplement: Supplementary file 6 [file Image_6.tif]

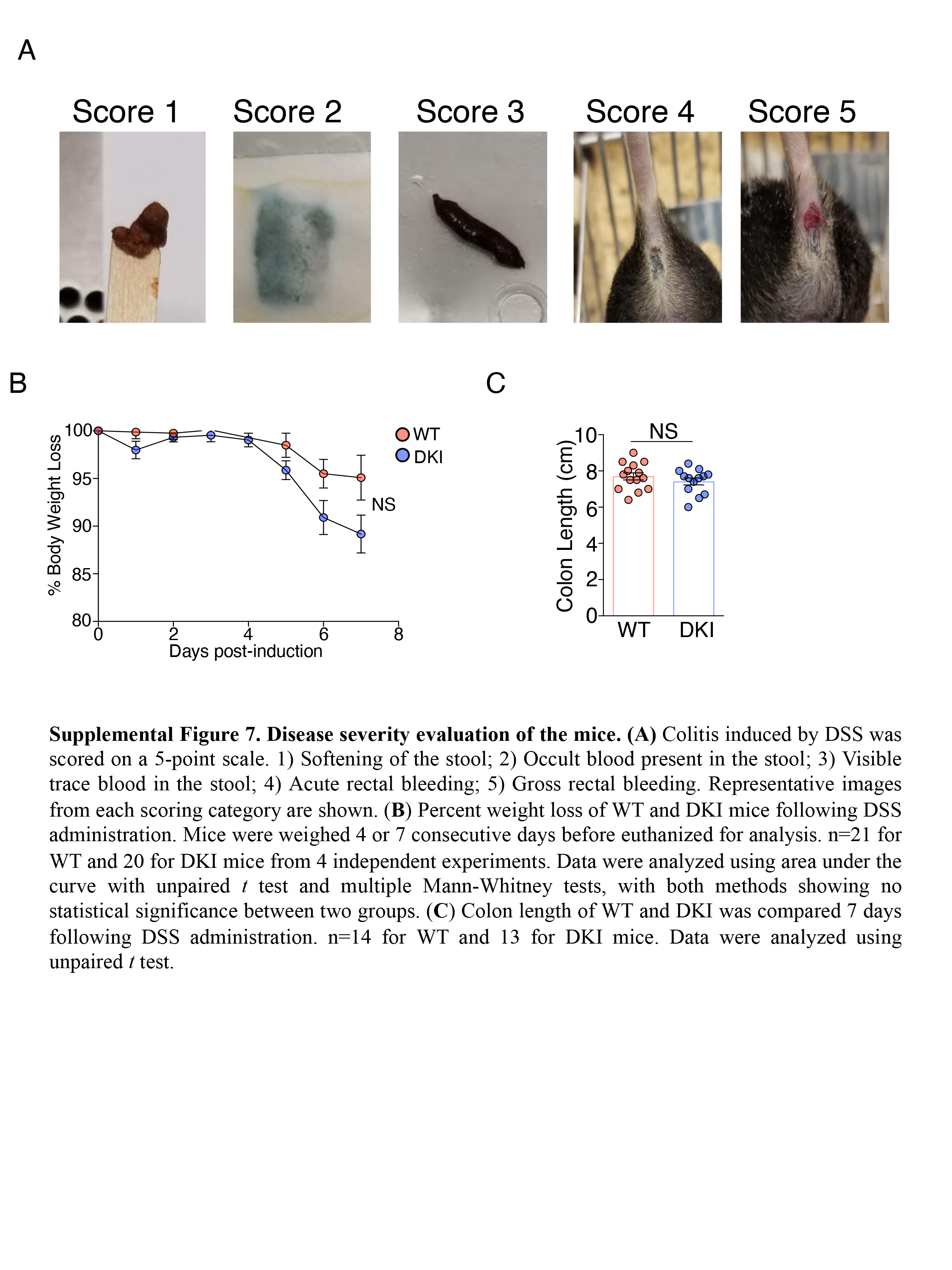

Supplement: Supplementary file 7 [file Image_7.tif]

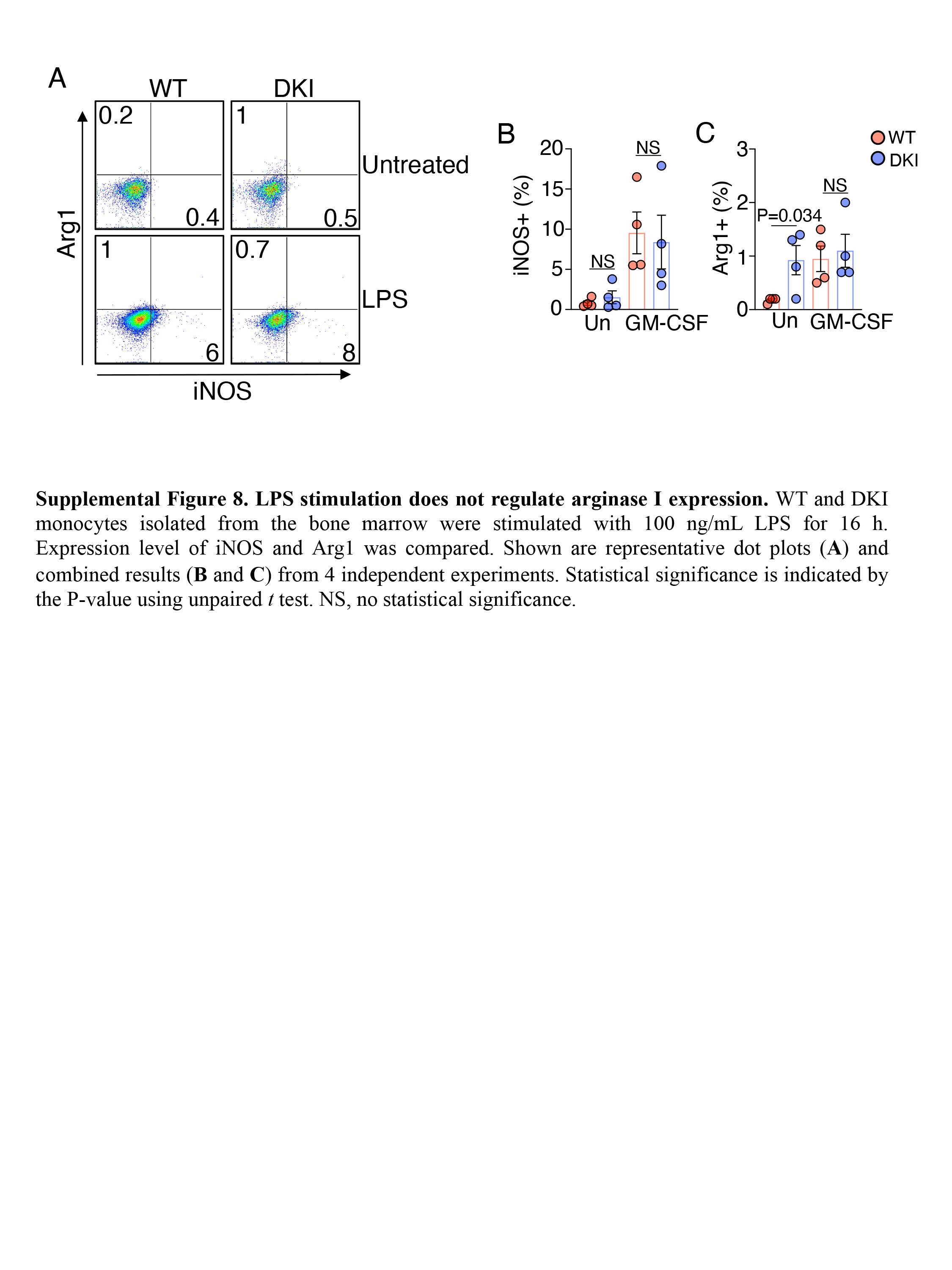

Supplement: Supplementary file 8 [file Image_8.tif]
